# Reproductive potential of the male (RPM): the computer database of phenotypic and molecular genetic data for Russian men with impaired and normal fertility

**DOI:** 10.1515/jib-2020-0032

**Published:** 2020-12-14

**Authors:** Anton Bogomolov, Alexander Osadchuk, Ludmila Osadchuk

**Affiliations:** The Federal Research Center Institute of Cytology and Genetics SB RAS, 630090, pr. Lavrentyeva 10, Novosibirsk, Russia, http://www.bionet.nsc.ru/en; Novosibirsk State University, 630090, Pirogova str., 1, Novosibirsk, Russia, https://english.nsu.ru/

**Keywords:** database, male fertility, phenotypic and molecular-genetic markers, spermatogenesis

## Abstract

Currently, a trend of decline in male fertility is observed all over the world. The study of this trend has not only of scientific, but also of socio-economic importance. Many countries conduct studies of male reproductive potential and search environmental and genetic causes of the mentioned phenomenon. Previously published studies have not included data on the Russian male population. This work presents novel database “Reproductive Potential of the Male population of Russia” (RPM), which is almost the only source of such information about Russia. RPM was created using relational database management system MariaDB and is available at www.sysbio.ru/rpm. The database includes reproductive information of 1390 male volunteers from five large cities of Russia: Arkhangelsk, Novosibirsk, Kemerovo, Ulan-Ude and Yakutsk. The important feature of the developed database is the unique data of a multifactorial measurement of male fertility: spermatogenic, hormonal, metabolic and anthropological indices. The scientists can use published information in their studies of male reproductive potential as data of some Russian regions and compare it with data from other countries. Also the published data can be used to identify markers of infertility and subfertility, as well as to study ethnic and regional trends in fertility variability and demographic risks in Russia.

## 1 Introduction

Over a past few decades, the declining trend of male reproductive function is observed in different regions of the world. It is reflected in a decreased quality of sperm, increased male factor in infertility couples and incidence of congenital abnormalities of the male reproductive system leading to infertility [1], [2]. According to a rough estimate, 5–7% of men suffer from the infertility disorders, while in about half of cases spermatogenesis defects are diagnosed.

Given the increasing socio-economic risks associated with population reproduction, studies evaluating male factor infertility are conducted in various countries in the world (e.g., Denmark, Norway, Estonia, Finland [3], USA [4], Estonia, Latvia, Lithuania [5], Norway [6], Russia [7], India, Thailand, Israel, China, Saudi Arabia, Japan, Korea [8]). One of the research outputs is a significant regional and ethnic difference in sperm production and quality and the profile of reproductive hormones. Thus, the region of residence and ethnicity are important determinants of male reproductive potential.

In Russia, the study of regional and ethnic differences in male reproductive potential began relatively recently, although information about regional and ethnic variability of male fertility in Russia is important for global trends analysis.

The male reproductive potential can be indirectly estimated using the Human Fertility Database [9] and reports of Federal State Statistic Service [10]. These resources contain information about total number of live births, crude birth rates, total fertility rates, mean age of men and so on. However, the above parameters do not give a picture of men infertility. It requires not only birth and population statistic, but also physiological and biochemical characteristics of the male reproductive potential (quantity indicators of ejaculate, anthropometric measurements and so on).

Notice that the research within the field of infertility has placed a disproportionate emphasis on the female component of reproduction. This is partly due to women infertility is more often diagnosed. As a result, the field of male infertility severely lacks high quality, large-scale studies evaluating important questions as they relate to male fertility. The same is true for male fertility databases. Their number is a handful. They are usually regional and do not confirm the uniform standard of presenting information [11]. The data sources for databases are questionnaires of men and health care information (hospital records, surgery records and outpatient visits). It should be noted, that databases based on medical records include information from patients, who sought infertility treatment (e.g., Truven Health MarketScan Databases [12], Utah Population Database [13]).

The RPM database is practically the only open source of male fertility in Russia. It contains phenotypic markers of male volunteers from five cities of the Russian Federation. Further expansion of the database is planned by including molecular genetic markers. An important feature of the database is multi-factorial fertility assessment of persons with normal and impaired spermatogenesis.

## 2 Materials and methods

The database contains data, obtained during 13 trips (research trials) to five Russian towns (Arkhangelsk, Novosibirsk, Kemerovo, Ulan-Ude, Yakutsk). The towns were selected based on their geographic location and ethnic composition of the population. This choice allows assessing the influence of the environment and ethnic differences on the semen quality.

The formation of a representative sample of male volunteers was based on the method of random samples from the general population. All participants gave informed consent to participation in the examination. The physical examination includes questionnaires, anthropometrical examination by the andrologist, results of semen analysis, enzyme immunoassay for inhibin B, testosterone, follicle-stimulating hormone and luteinizing hormone. The database captures information from questionnaire regarding age, ethnic and racial background, lifestyle factors, previous or current urological diseases, history of fertility and other information that could influence on reproductive functions.

The andrologist examined each men-volunteer including anthropometry, measurement of arterial pressure, determining testicular volume, examining the external genitalia, collecting history and preliminary andrological diagnosis.

Semen samples were analyzed for semen volume (mL), sperm concentration (mln/mL), progressive motility (percentage) and normal morphology (percentage) according to the WHO guidelines for the examination of human semen [14]. The level of spermatozoa DNA fragmentation was estimated by method of sperm chromatin structure assay [15].

The serum concentrations of reproductive hormones were determined by enzyme-linked immunosorbent assay [16]. The blood analysis also included measuring the concentrations of triglycerides, total cholesterol, glucose, uric acid, high-density and low-density lipoprotein cholesterol. The above mentioned parameters were determined by the enzymatic colorimetric method.

The detection of microdeletions in the Azoospermia Factor (AZF) locus of the Y chromosome and the calculation the number of CAG repeats in the first exon of the androgen receptor gene is performed for all men-volunteers at present time. Identification of microdeletions and determination of the number of CAG repeats in the gene are performed by fragment analysis using the existing nanofor05 capillary sequencer based on Sequence-Tagged Sites (STS) markers (Devyser AZF v2 RUO and Devyser AZF Extension RUO sets) and tested primers [17] respectively.

The ethics committee of the Federal Research Center ‘Institute of Cytology and Genetics’, the Siberian Branch of the Russian Academy of Sciences approved the study (see the supplementary).

## 3 Results

**Figure 1:**
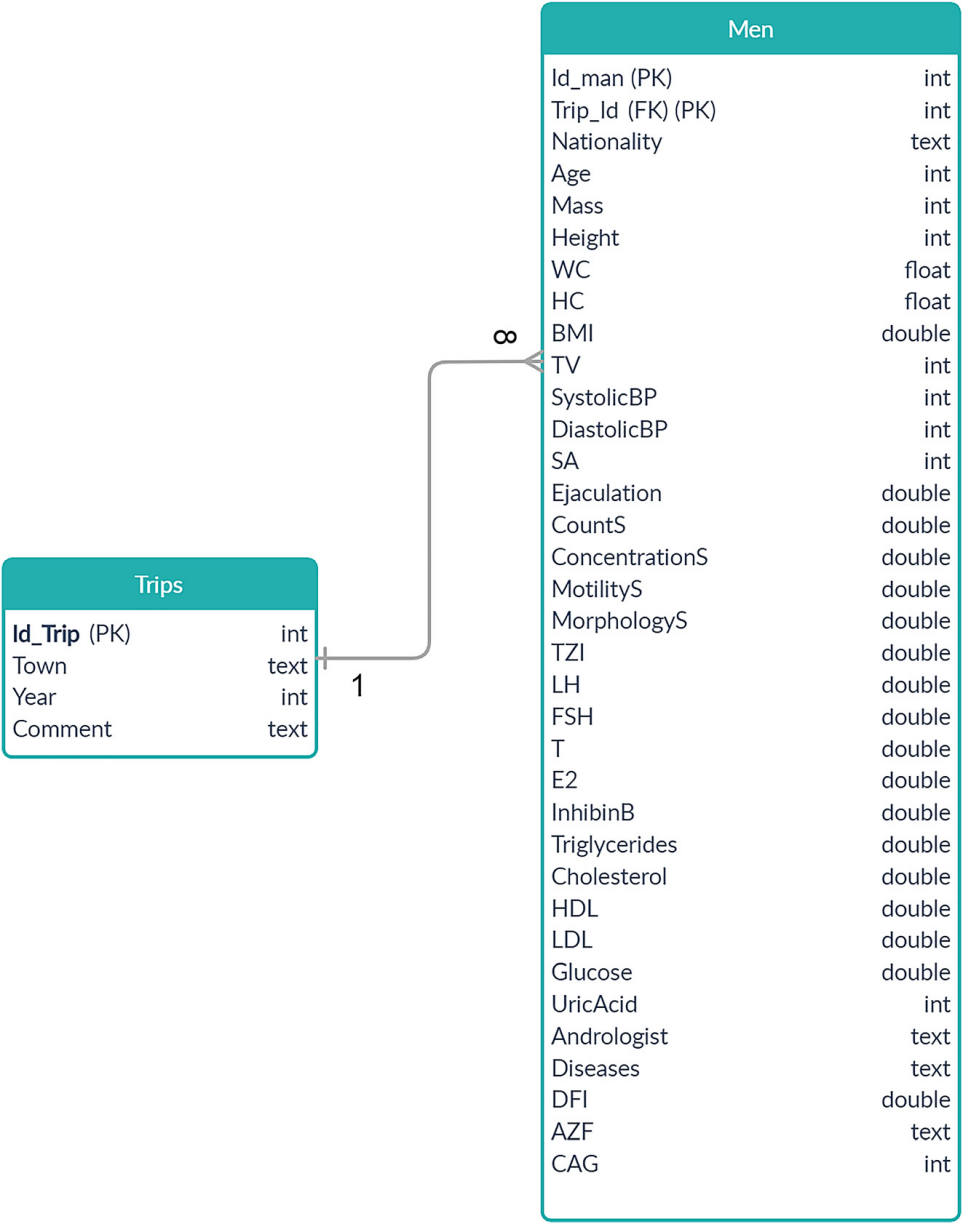
General scheme of the database «Reproductive potential of the male population of Russia» (ER-model of the database). The primary and foreign keys are marked PK and FK, accordingly.

The computer database of phenotypic data of men with impaired and normal spermatogenesis was created using an open-source relational database management system MariaDB 10.2.12 (MariaDB Corporation AB, https://mariadb.org/). This database was named “Reproductive Potential of the Male population of Russia” (RPM). It consists of two tables: “Trips” and “Men” (Figure 1 shows scheme of the database).

The “Trips” table contains information about the trips, during which the phenotypic material was obtained. Each trip is described with two parameters: the year of the trip (“Year”) and the destination city (“Town”).

The table “Men” contains phenotypic data of men. It includes 32 markers of male reproductive potential and three markers will be added soon. More detailed information of the individual markers is presented in Table 1.

**Table 1:** Description of the columns in the Table “Men”, associated with fertility.

Field (marker)	Description	Type of information
Age	Age of the volunteer	Data from questionnaire survey
Andrologist	Preliminary andrologic diagnosis	Anthropometrical examination
AZF	Type of microdeletions in the AZF locus of the Y chromosome. This marker will be added	Genetic markers
BMI	Body mass index	Anthropometrical examination
CAG	Number of CAG repeats in the first exon of the androgen receptor gene. This marker will be added	Genetic markers
Cholesterol	Total cholesterol	Metabolic indices
ConcentrationS	Sperm concentration	Semen parameters
CountS	Total number of spermatozoa	Semen parameters
DFI	Level of spermatozoa DNA fragmentation (DNA fragmentation index). This marker will be added.	Semen parameters
DiastolicBP	Diastolic blood pressure	Anthropometrical examination
Diseases	Infertility history, lifestyle factors, chronic disease, venereal disease and occupational hazards	Data from questionnaire survey
E2	Estradiol	Hormonal indices
Ejaculation	Ejaculate volume	Semen parameters
FSH	Follicle-stimulating hormone	Hormonal indices
Glucose	Glucose	Metabolic indices
HC	Hip circumference	Anthropometrical examination
HDL	High density lipoproteins	Metabolic indices
Height	Height	Anthropometrical examination
Inhibin B	Inhibin B	Hormonal indices
LDL	Low density lipoproteins	Metabolic indices
LH	Luteinizing hormone	Hormonal indices
Mass	Body weight	Anthropometrical examination
MorphologyS	Percent of morphologically normal spermatozoa	Semen parameters
MotilityS	Percent of motile spermatozoa	Semen parameters
Nationality	National roots	Data from questionnaire survey
SystolicBP	Systolic blood pressure	Anthropometrical examination
T	Testosterone	Hormonal indices
Triglycerides	Triglycerides	Metabolic indices
TV	Bitesticular volume	Anthropometrical examination
TZI	Ratio of the number of morphological defects to the number of defective spermatozoa	Semen parameters
UricAcid	Uric acid	Metabolic indices
WC	Waist circumference	Anthropometrical examination

**Figure 2:**
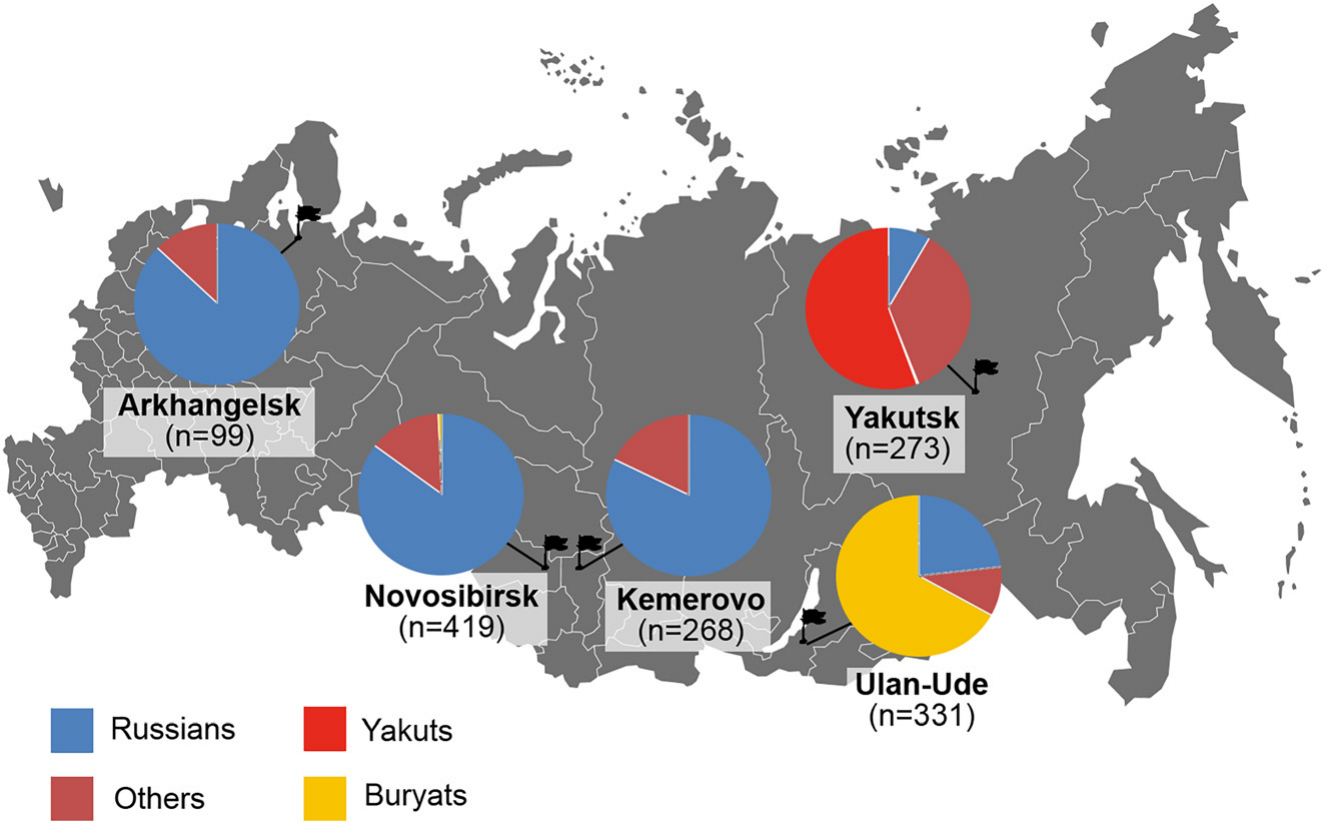
Geographic and ethnic distribution of male volunteers. *n*- the number of men in the sample. The others are men, who identify themselves as descendants from mixed marriages between people of different nationalities or representatives of national (ethnic) minorities (less than five percent in the sample of the corresponding city).

Now the database includes reproductive information of 1390 male volunteers from five large cities of Russia, located in the regions with different climatic conditions, environmental situation and national composition of the population: Arkhangelsk, Novosibirsk, Kemerovo, Ulan-Ude and Yakutsk (Figure 2).

As illustrated (in Figure 2), three ethnic groups are mainly represented in the selected samples of men: Slavs, Buryats and Yakuts. The mean value and standard deviation of age of the men during the study period was 25.4 ± 7.5. The youngest person was 18 years old and the oldest was 63 years.

**Figure 3:**
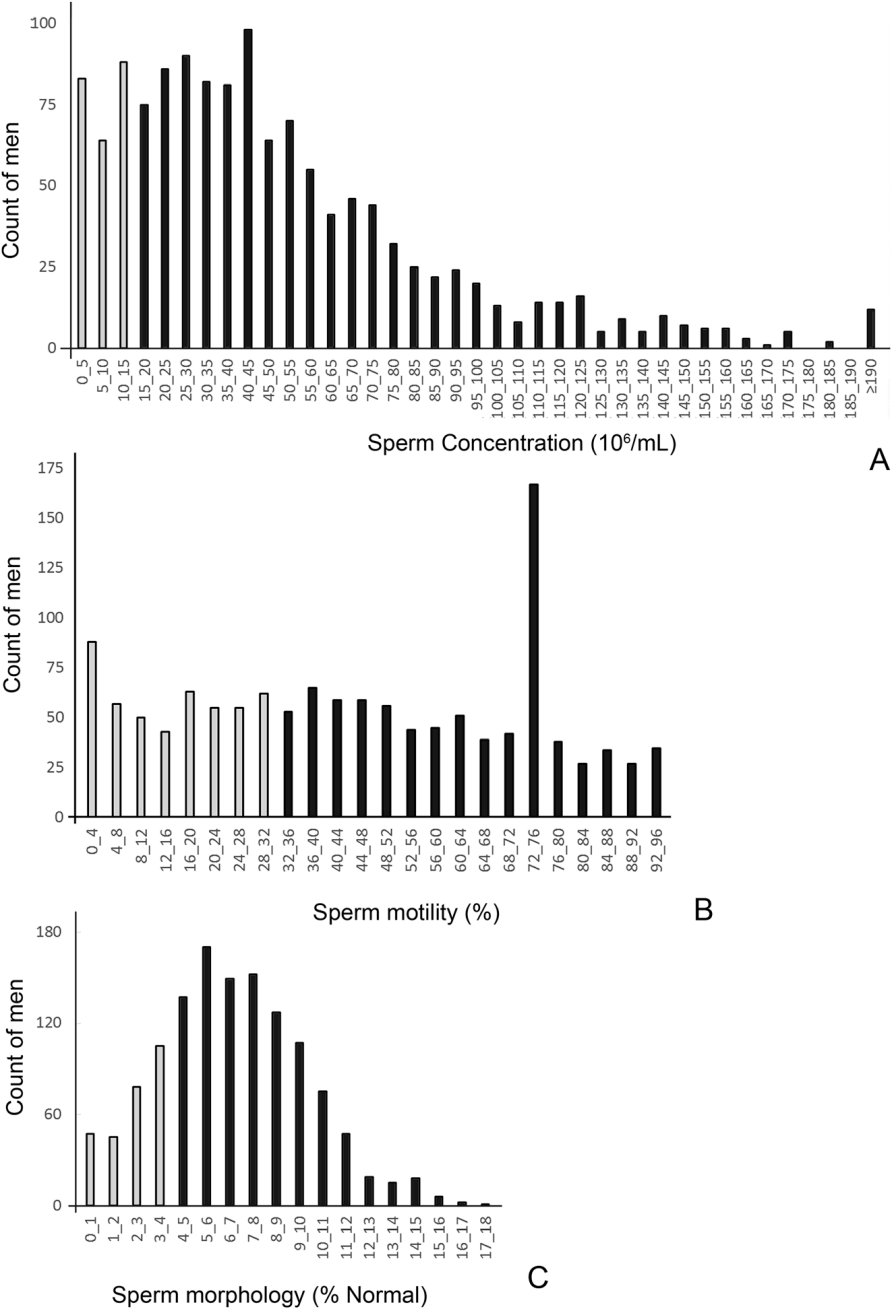
Frequency distribution of basic semen parameters of men-volunteers. A – 17.7% of cases have oligozoospermia with sperm concentration below 15 × 10^6^/mL. B – 36% have asthenozoospermia with less than 32% motile spermatozoa. C – 21.2% have teratozoospermia with less than 4% morphologically normal spermatozoa. Gray columns indicate the percentage of men with values below the World Health Organization thresholds.

Comparing with the reference level set by the WHO for fertile men, 17.7% of men had sperm concentration below 15 Mio/mL (Figure 3A). In terms of motility, 36% displayed low values with less than 32% motile sperms and 21.2% had less than 4% morphologically normal sperm cells (Figure 3B, C).

So the database contains information about 664 men with normospermia (54.7%), 662 men with different abnormalities of semen quality (40.6%) and 64 peoples with incomplete data of semen analysis (4.6%) (more detailed distribution of men is shown in Table 2).

**Table 2:** Distribution of men according to their semen characteristics in the period 2009–2014 in the European North and Siberian regions of Russia.

Semen parameters	Absolute number							%
	2009	2010	2011	2012	2013	2014	Total	
Normozoospermia	195	214	139	40	62	111	761	54.7
Asthenozoospermia	46	57	32	20	17	19	191	13.7
Oligoasthenoteratozoospermia	21	50	23	4	16	9	123	8.8
Asthenoteratozoospermia	11	32	15	10	5	8	81	5.8
Oligoasthenozoospermia	21	24	15	5	7	6	78	5.6
Teratozoospermia	17	19	13	3	4	1	57	4.1
Oligozoospermia	11	4	4	2	0	0	21	1.5
Oligoteratozoospermia	4	3	6	0	0	1	14	1
Unknown	36	9	15	1	1	2	64	4.6
Total	362	412	262	85	112	157	1390	100

**Figure 4:**
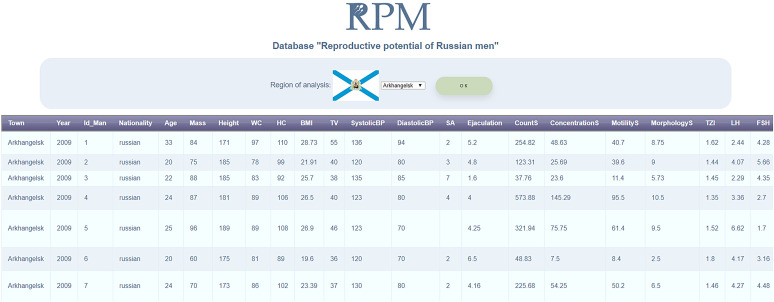
Example of the web-interface to the database «Reproductive potential of the male population of Russia». The visualization screen shows example of data from the Arkhangelsk city.

The database is available at www.sysbio.ru/rpm/. Currently the web-interface allows users to observe collected data and use simple filters (select data by city, by nationality groups, by period of trips etc.). The example of data visualization is shown in Figure 4.

## 4 Discussion

The «Reproductive potential of the male population of Russia» is the database specifically designed for analyzing male reproductive potential. The important feature of the developed database is the unique results of a multifactorial measurement of male fertility. Moreover, it contains a lot of additional information (metabolic, anthropological indices, ethnic and racial background, lifestyle factors and so on) that allows us to research associations between this information and the markers of infertility. The three markers will be added soon: the level of spermatozoa DNA fragmentation, the detection of microdeletions in the AZF locus of the Y chromosome and the calculation the number of CAG repeats in the first exon of the androgen receptor gene. It is a known fact that the increased level of sperm DNA fragmentation is associated with infertility, low fertilization level, miscarriage in a married couple, which determines the importance of DNA fragmentation index both in the diagnosis of infertility and in population studies of male fertility [18]. The microdeletions of long arm Y-chromosome are the most common genetic cause of male infertility. This locus of the Yq euchromatin (locus Yq11.21–23) contains three sub regions: AZFa, AZFb and AZFc, the complete deletions of which lead to azoospermia and severe oligospermia. The androgen receptor influences on process of sexual differentiation and normal spermatogenesis regulation. It is well known that anomaly count of CAG repeats in the first exon of this gene increases the risk of spermatogenesis defects and infertility [19]. For this reason, those markers are important indicators of reproductive disorders in the clinical diagnostic.

The published data were obtained according to the uniform protocol of clinical and physiological parameters of male fertility. This allows studying of the male population of the European North and Siberian regions of Russia (an example of such an analysis is shown in Figure 2) or groups of data, combined based on different characteristics (by region, by nationality groups, by age etc.).

It should be noted, that the chosen towns differ in climatic conditions: temperate climate, excessive soil moisture conditions (Arkhangelsk); continental climate, taiga and forest-steppe with sufficient soil moisture conditions (Novosibirsk, Kemerovo); extremely continental climate, taiga with changeable soil moisture conditions (Ulan-Ude, Yakutsk). Moreover, they differ in natural and man-made background of the ecological situation. Kemerovo is a region with a high level of air and soil pollution in the period from 2007 to 2016 [20], [21]. The influence of environmental factors on male fertility may be a subject for the further research.

Another feature of the database RPM is a large part of data from healthy young men from the general population (Table 2). Very often patients seeking health care provide the information about sperm quality in similar databases [22].

While the RPM has many clear strength, it suffers some flaws. Similar to other fertility databases, the retrospective nature of the data can be limiting for certain studies. The data cover the period from 2009 to 2014 years. Additionally, the populations included in the RPM live in the Arkhangelsk area, Kemerovo area, Novosibirsk area, the Sakha Republic, the Republic of Buryatia, but this study population is likely unrepresentative the whole Russian population and its other regions, taking into account its vast territory and multinational composition. In this connection, the current database will be complemented with data obtained during new trips in other cities of Russia.

In order to indicate the underlying causes, extensive research has been done on the genetic reasons of male infertility in recent years. A number of polymorphisms that have a fundamental role in sperm chromatin density and spermatogenesis have been empirically discovered [23]. The results of the RPM can serve as a basis for further prospective studies of the molecular genetic mechanisms of pathogenesis of reproductive diseases, determining the prognosis of treatment efficacy, and developing new approaches to personalized treatment of genetic disorders leading to infertility. Furthermore, it is planned to extend the RPM to include information about whole-exome sequencing (WES) data from men with impaired and normal spermatogenesis. A study using next-generation sequencing in our large cohorts of patients from the general population that includes subfertile, infertile, and fertile men could provide interesting new data on the ethnic prevalence of genes involved in poor reproduction, and improve diagnostic tests. The use of our phenotypic database RPM will allow you to use the approach based on phenotype to identify new genes.

## 5 Conclusion

The database “Reproductive potential of the male population of Russia” has been developed, based on the collected reproductive information of male volunteers from five cities of Russia (Arkhangelsk, Novosibirsk, Kemerovo, Ulan-Ude and Yakutsk). It contains unique phenotypic and molecular-genetic data, characterizing the reproductive potential of male populations in the cities listed above. The published data can be used to identify markers of infertility and subfertility, as well as to study ethnic and regional trends in fertility variability and demographic risks in Russia. The database developed provides information for comparative study of male reproductive potential in different regions.
