# Supplementary material for: Reproductive potential of the male (RPM): the computer database of phenotypic and molecular genetic data for Russian men with impaired and normal fertility
Source: J Integr Bioinform. 2020 Dec 14;17(4):20200032. doi: 10.1515/jib-2020-0032 (PMC7790180; doi:10.1515/jib-2020-0032)
Supplement: Supplementary file 1 [file jib-17-20200032-s001.pdf]

Научно-исследовательский институт
клинической и экспериментальной лимфологии –
филиал Федерального государственного бюджетного научного учреждения
«Федеральный исследовательский центр Институт цитологии и генетики
Сибирского отделения Российской академии наук"
(НИИКЭЛ – филиал ИЦиГ СО РАН)

Ул. Тимакова, 2,
Новосибирск, 630060
телефон (383) 333-64-09
E-mail: Lek@niikel.ru
ОКПО 15769226
ОГРН 1025403657410
ИНН 5408100138
КПП 540843001

Выписка из протокола N160
заседания этического комитета НИИКЭЛ – филиал ИЦиГ СО РАН
17 сентября 2020г.

Присутствовали члены этического комитета: Летягина Е.А. -председатель, Бгатова Н.П., Бурштейн Е.С., Евсеева С.М., Королева Е.А., Миронов В.А., Хабаров Д.В., Шемякина Н.Н.- секретарь.
Заседание состоялось в помещении клиники НИИКЭЛ – филиал ИЦиГ СО РАН
17.09.2020 в 14:00 часов по адресу: г. Новосибирск, ул. Арбузова, 6.

III. Слушали: об одобрении проведения научно-исследовательской работы: «Популяционное исследование мужской фертильности урбанизированного населения Российской Федерации: оценка возможностей полно-экзомного секвенирования и идентификация новых генов, ассоциированных с ослабленным сперматогенезом» на базе Федерального государственного бюджетного научного учреждения «Федеральный исследовательский центр Институт цитологии и генетики Сибирского отделения Российской академии наук». Научный руководитель: к.б.н., доц. Осадчук Александр Владимирович.

Заявитель: к.б.н., доц. Осадчук Александр Владимирович.

На рассмотрение предоставлены следующие документы:

1. Протокол клинического исследования: «Популяционное исследование мужской фертильности урбанизированного населения Российской Федерации: оценка возможностей полно-экзомного секвенирования и идентификация новых генов, ассоциированных с ослабленным сперматогенезом».
2. Информированное согласие на участие в исследовании.

Приняли решение:

1. При проведении этической экспертизы по представленным материалам замечаний нет.

2. Одобрить проведение научно-исследовательской работы: «Популяционное исследование мужской фертильности урбанизированного населения Российской

Федерации: оценка возможностей полно-экзомного секвенирования и идентификация новых генов, ассоциированных с ослабленным сперматогенезом» на базе Федерального государственного бюджетного научного учреждения «Федеральный исследовательский центр Институт цитологии и генетики Сибирского отделения Российской академии наук»

Проголосовали: «за» - 8 человек, «против» -0, «воздержавшихся» -0.

Председатель ЛЭК Е.А. Летягина

Секретарь ЛЭК Н.Н. Шемякина
